# Slit and Netrin-1 guide cranial motor axon pathfinding via Rho-kinase, myosin light chain kinase and myosin II

**DOI:** 10.1186/1749-8104-5-16

**Published:** 2010-06-22

**Authors:** Ailish Murray, Arifa Naeem, Sarah H Barnes, Uwe Drescher, Sarah Guthrie

**Affiliations:** 1 MRC Centre for Developmental Neurobiology, 4th Floor New Hunt's House, King's College, Guy's Campus, London SE1 1UL, UK

## Abstract

**Background:**

In the developing hindbrain, cranial motor axon guidance depends on diffusible repellent factors produced by the floor plate. Our previous studies have suggested that candidate molecules for mediating this effect are Slits, Netrin-1 and Semaphorin3A (Sema3A). It is unknown to what extent these factors contribute to floor plate-derived chemorepulsion of motor axons, and the downstream signalling pathways are largely unclear.

**Results:**

In this study, we have used a combination of *in vitro *and *in vivo *approaches to identify the components of floor plate chemorepulsion and their downstream signalling pathways. Using *in vitro *motor axon deflection assays, we demonstrate that Slits and Netrin-1, but not Sema3A, contribute to floor plate repulsion. We also find that the axon pathways of dorsally projecting branchiomotor neurons are disrupted in *Netrin-1 *mutant mice and in chick embryos expressing dominant-negative *Unc5a *receptors, indicating an *in vivo *role for Netrin-1. We further demonstrate that Slit and Netrin-1 signalling are mediated by Rho-kinase (ROCK) and myosin light chain kinase (MLCK), which regulate myosin II activity, controlling actin retrograde flow in the growth cone. We show that MLCK, ROCK and myosin II are required for Slit and Netrin-1-mediated growth cone collapse of cranial motor axons. Inhibition of these molecules in explant cultures, or genetic manipulation of RhoA or myosin II function *in vivo *causes characteristic cranial motor axon pathfinding errors, including the inability to exit the midline, and loss of turning towards exit points.

**Conclusions:**

Our findings suggest that both Slits and Netrin-1 contribute to floor plate-derived chemorepulsion of cranial motor axons. They further indicate that RhoA/ROCK, MLCK and myosin II are components of Slit and Netrin-1 signalling pathways, and suggest that these pathways are of key importance in cranial motor axon navigation.

## Background

Developing axons are guided by a range of molecules, including diffusible chemorepellents [1-3]. In the developing vertebrate hindbrain, the initial trajectory of cranial motor axons depends on repulsion from the ventral midline floor plate [4]. Both dorsally projecting branchiomotor and visceral motor (BM/VM) neuron subtypes and ventrally projecting somatic motor (SM) neuron subtypes respond to floor plate repulsion.

*In vitro *experiments have shown that the diffusible chemorepellents Netrin-1, Slit-1 and -2 and Semaphorin3A (Sema3A) repel BM/VM axons, whereas SM axons respond only to Sema3A [5-7]. Both Netrin-1 and Slits, but not Sema3A, are expressed by the hindbrain floor plate at times of early motor axon extension [7-11]. Slit-2 is also expressed by the rhombic lip, which borders BM/VM axon pathways dorsally [7,12]. Thus, Netrin-1 and the Slits are prime candidates to mediate BM axon repulsion *in vivo*. Our hypothesis is that floor plate repulsion drives BM axons away from the midline, whereas a dorsal domain of repulsion might 'hem them in' to dorsal exit points. Consistent with this idea, BM neurons express *Unc5a *(also known as *UNC5H1*) and *Robo1/2 *receptors, which are required to mediate the repellent effects of Netrin-1 and Slits, respectively [7,13-15]. Whereas a role for Slit-Robo signalling in cranial motor axon repulsion has been demonstrated *in vivo *[7], the extent of Netrin-1's contribution has been less clear. In this study, we therefore first examined the possible role of Netrin-1, and then determined the relative contribution of the putative molecular components of floor plate repulsion.

We have also investigated the signalling pathways involved in BM/VM (which we will now refer to as BM) axon guidance decisions, of which little is known. A key process in repellent growth cone decisions is actin retrograde flow, driven by myosin molecular motors, and in particular, myosin II (reviewed by [16]). Myosin II function is positively regulated by the phosphorylation of its regulatory light chain (MRLC) by myosin light chain kinase (MLCK) and negatively regulated by myosin light chain phosphatase. Both MLCK and RhoA kinase (ROCK) phosphorylate MRLC, while ROCK also indirectly activates myosin II by inhibiting myosin light chain phosphatase. Thus, RhoA acts via ROCK to control actin retrograde flow [17,18]. However, there is as yet no evidence to suggest that myosin II operates downstream of Netrin-1 or Slits, and it has not been shown to play a role in vertebrate axon pathfinding *in vivo*.

In this study we have used a floor plate-motor axon deflection assay, and present evidence that both Netrin-1 and Slit chemorepellents contribute to BM axon deflection. We also analysed *Netrin-1 *mutant mice [19], and chick embryos in which a dominant negative form of the Netrin receptor Unc5a was electroporated into cranial motor neurons. In both cases cranial motor axon trajectories were altered in a manner consistent with a role for Netrin-1 in their pathfinding. We then used a combination of *in vitro *assays and *in vivo *electroporations in chick embryos to investigate whether Netrin-1 and Slit signalling in cranial motor neurons depends on ROCK, MLCK and myosin II. Inhibitors of ROCK, MLCK or myosin II abrogated or strongly attenuated cranial motor neuron growth cone collapse, and produced pathfinding errors in explant cultures. Electroporation of dominant-negative forms of RhoA and MRLC, and of a constitutively active form of MRLC also resulted in cranial motor axon pathfinding defects *in vivo*. Taken together, these data suggest that cranial motor axon repulsion by Slit and Netrin-1 is of crucial importance in governing axon pathfinding in the hindbrain, and is mediated by ROCK, MLCK and myosin II.

## Results

### Netrin-1 contributes to floor plate-mediated repulsion *in vivo*

**Figure 1:**
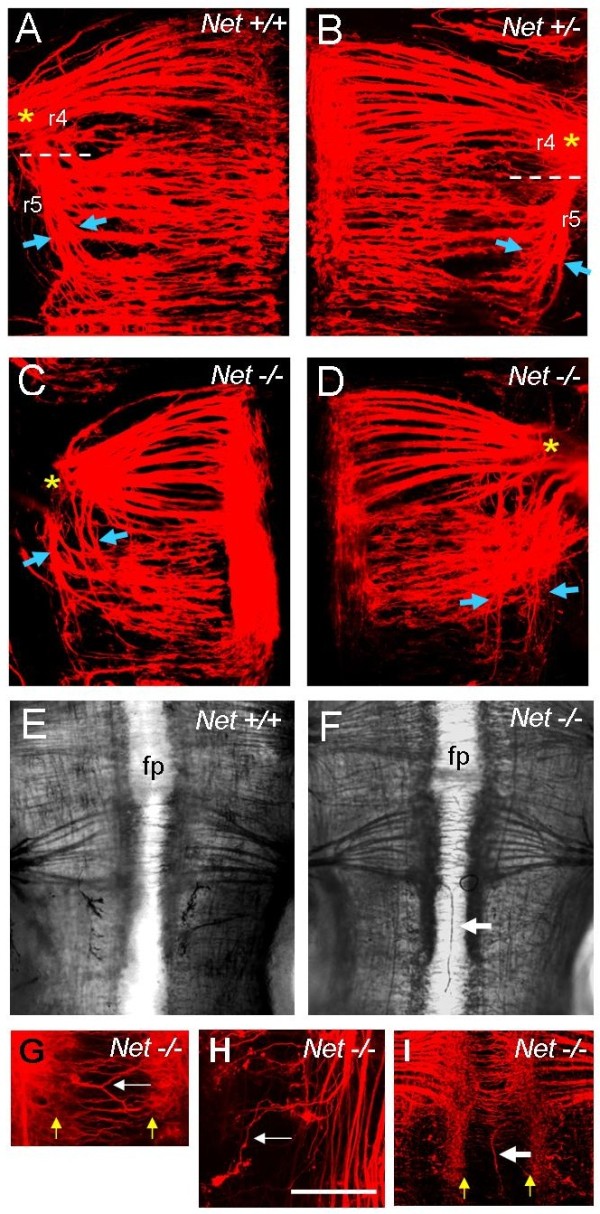
***Netrin-1 *mutants show cranial motor axon guidance defects**. E11.5 mouse hindbrains from wild-type, heterozygous and homozygous *Netrin-1 *mutants; genotypes as indicated. **(A-D) **Facial motor neurons in r4 and r5 retrogradely labelled using DiI. Rhombomeres 4 and 5 are numbered in (A) and (B); asterisks indicate r4 exit points. Arrows indicate width of fascicles growing towards exit points. Note increased width and defasiculation of axon bundles in homozygotes, with many separate axon bundles near the exit points. **(E,F) **Wholemount anti-neurofilament staining of motor neurons in E11.5 mouse hindbrains. Note longitudinal axon fascicle in homozygote (arrow in (F)). **(G-I) **Motor axon pathfinding defects in DiI-labelled hindbrains of Netrin mutant mice. Yellow arrows indicate boundaries of the floor plate, and white arrows indicate defects. (G) Axon fascicles projecting at an angle rather than crossing the floor plate directly. (H) Axon turning prematurely towards exit point. (I) Fascicle projecting longitudinally within floor plate. fp, floor plate. Scale bar in (H) = 150 μm in (A-D,G-I) and 300 μm in (E,F).

In order to determine whether Netrin-1 guides BM axon projections, we analysed embryonic day 11.5 (E11.5) mice deficient in *Netrin-1 *function [19] using whole-mount immunostaining or retrograde axon tracing from the motor exit points. Taking facial motor neurons as an example, in wild-type embryos cranial motor axons within rhombomere 4 (r4) projected directly laterally towards their r4 exit point, whereas those within r5 projected laterally, and then turned rostrally to project towards their r4 exit point. Wild-type motor axon tracts were therefore organised into tight fascicles *en route *to their r4 exit points (Figure 1A). In heterozygous embryos, BM axons showed some evidence of turning prematurely towards the exit point and formed a wider fascicle (Figure 1B). In homozygotes this phenotype was more obvious, with the axon tracts separated into many fascicles (Figure 1C, D). Individual DiI-labelled motor axons could also be seen turning prematurely rostrally (Figure 1H). As only the floor plate region expresses Netrin-1 in the hindbrain at this developmental stage [20], this phenotype is most readily explained by a loss of repulsion of BM axons as they grow dorsally, so that some axons' rostral projection is premature relative to those in wild-type embryos. Anti-neurofilament immunostaining showed that in homozygous embryos, and with a lower frequency in heterozygous embryos, axons in r4 and r5 formed a longitudinal fascicle within the floor plate (Figure 1E, F; Table 1). These aberrant axonal pathways were also seen when motor neurons were retrogradely labelled using DiI, confirming their identity (Figure 1I; Table 1). At r4 level, where contralateral vestibuloacoustic neurons cross the midline, these axons diverted from their circumferential pathways to project for short distances longitudinally within the floor plate (Figure 1G).

**Table 1:** Axon guidance phenotypes in *Netrin-1 *mutant mice

Axon guidance defect	**Netrin-1 **+/+	Netrin-1 +/-	Netrin-1 -/-
Early axon turning to exit point	0/5	3/14	9/10
Rhombomere 4 fascicles projecting longitudinally short distances in floor plate	0/5	1/14	4/10
Long axon fascicle in floor plate	0/5	4/14	5/10

Data based on a combination of neurofilament immunostaining and DiI labelling.

These midline defects resemble those seen in mice deficient in either Slits or Robos [7], and point to a loss of repulsion from the midline. Netrin-1 repulsion is therefore required to keep BM axons out of the floor plate, and to repel axons during their onward pathway towards their dorsal exit points. A previous study showed that trochlear motor neuron cell bodies (originating in r1) were ectopically located in the floor plate in *Netrin-1 *mutants [19]. We focussed our attention on r2 to r8 levels but failed to see any consistent misplacement of cranial motor neuron cell bodies.

**Figure 2:**
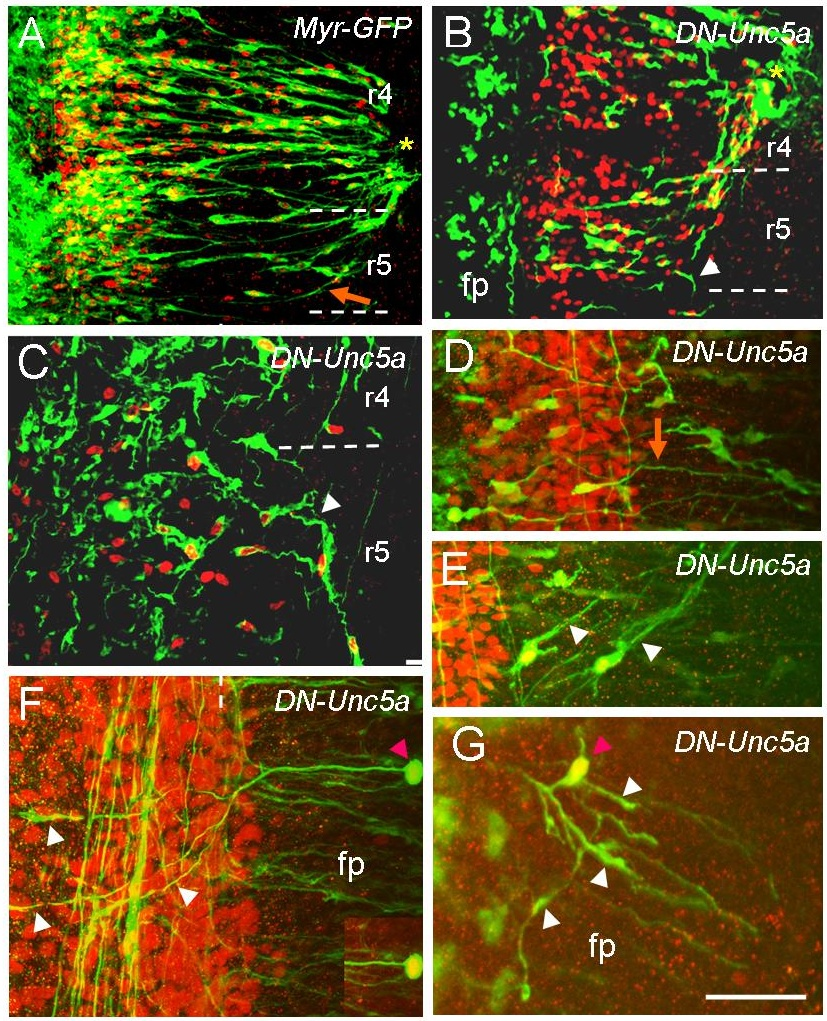
**Expression of dominant-negative forms of *Unc5a *in the chick hindbrain causes BM axon pathfinding errors**. (A-G) Flat-mount chick hindbrains that have been electroporated with plasmids encoding myristylated GFP (myr-GFP) or dominant-negative Unc5a (DN-Unc5a), as labelled. Immunostaining with anti-GFP (green) and anti-Islet1/2 (red) antibodies. In controls, axons in r5 grow rostrally and correctly towards exit point (orange arrow in (A)). In *DN-Unc5a*-expressing embryos some axons also pathfind normally (orange arrow in (D)), whereas some axons in r5 turn caudally (arrowheads in (B,C)). Some *DN-Unc5a*-expressing BM axons are orientated at an angle to the floor plate rather than perpendicularly (arrowheads in (E)). *DN-Unc5a*-expressing BM neuron cell bodies are found ectopically within the floor plate (pink arrowheads in (F,G)) and produce multiple branches (white arrowheads in (F,G)). Inset in (F) shows cell body with higher Islet-1/2 immunofluorescence. fp, floor plate. Scale bar: 50 μm (A,B); 25 μm (C-E); 20 μm (F,G).

As previous experiments had shown that cranial motor axons expressing dominant negative forms of Robo receptors for Slits show striking axon pathfinding defects [7], we expressed dominant negative forms of the Netrin receptor Unc5A in chick hindbrains. Such constructs would be expected to abrogate motor axon responses to Netrin-1. For this we used a bicistronic construct driven by the chick β-actin promoter with a cytomegalovirus enhancer, and containing an IRES-GFP. As a control, we electroporated a myristylated form of green fluorescent protein (GFP) in the same vector (myr-GFP) [7]. Chick embryo hindbrains were electroporated at stage 12/13, incubated for 48 hours, to stage 20/21, and immunostained for GFP and Islet-1/2 to identify electroporated motor neurons and their axon paths. BM axons expressing the control *myr-GFP *construct showed a preservation of normal axon paths, projecting a single axon perpendicularly to the floor plate, away from the motor column towards their dorsal exit points (Figure 2A). At facial level, for example, r4 BM axons projected towards their r4 exit point, whereas axons in r5 projected laterally, making a rostral turn to project to the r4 exit point. Many BM axons electroporated with dominant-negative *Unc5a *(*DN-Unc5a*) also projected in a superficially normal manner away from the midline and towards their exit points (Figure 2B,D). However, there was some disorder in the orientation of axon projections, and among normally projecting axons we observed some that failed to grow laterally, with some axons within r5 projecting caudally to r6 rather than rostrally to r4 (Figure 2B, C; 8 out of 12 embryos). Some axons were misaligned, projecting away from the motor column at an angle rather than perpendicular to the floor plate (Figure 2E). In 6 out of 12 electroporated embryos, we also noticed that *Unc5a*-expressing BM neuron cell bodies were present in the floor plate and neurons produced several branches (Figure 2F, G). The presence of ectopic BM neurons in the floor plate echoes phenotypes seen in DN-Robo-electroporated embryos [7], but in the latter case, ectopic branching was never seen. Overall, these phenotypes were less severe than those in DN-Robo-expressing embryos. Nevertheless, they suggest that Netrin-1 repulsion helps to specify the lateral projection of BM axons, and might additionally act to suppress inappropriate branch formation.

### Both Slit and Netrin contribute to floor plate-mediated repulsion *in vitro*

**Figure 3:**
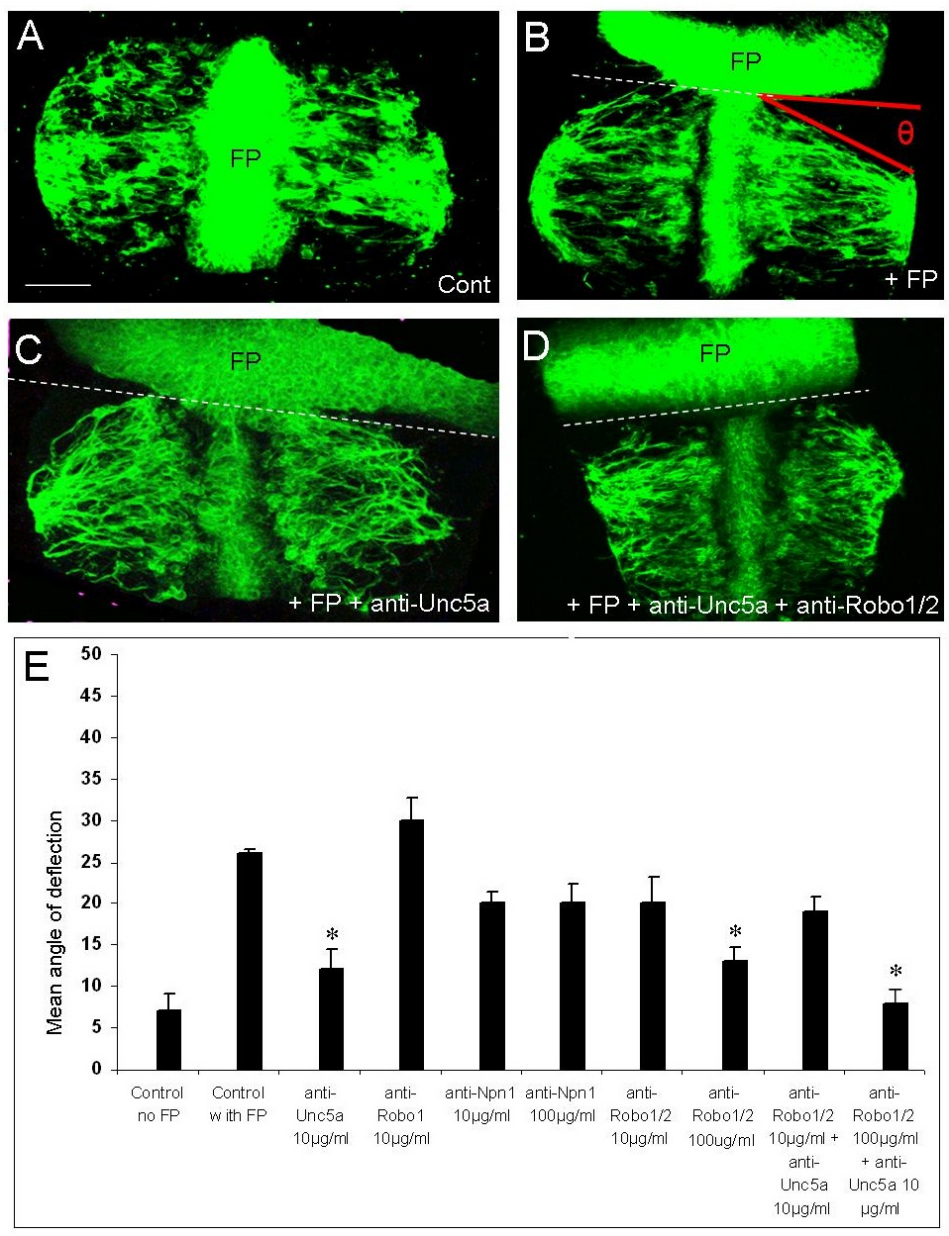
**Cranial motor axon repulsion is blocked by anti-Unc5a antibodies and/or anti-Robo1/2 antibodies in a floor plate deflection assay**. **(A) **Hindbrain control (Cont) explant containing the floor plate (FP) and BM neurons immunostained with anti-SC1 antibodies. **(B) **Hindbrain explant cultured with juxtaposed floor plate shows motor axonal deflection θ. **(C) **Application of anti-Unc5a antibodies reduces this deflection. **(D) **Application of anti-Unc5a + anti-Robo1/2 antibodies together reduces the deflection further. Dotted lines show floor plate-explant border. Scale bar = 100 μm. **(E) **Graph showing the mean angle of deflection in the presence of various antibodies (N = 40 to 50 explants in each category). Asterisks denote categories showing blockade of repulsion, that is, reduction of axon deflection to level of 'Control no FP'.

In order to determine the molecular components of floor plate repulsion, we used a deflection assay in which bilateral ventral third hindbrain explants (which contain an internal floor plate) were juxtaposed with a second floor plate explant at 90° in collagen gels and cultured for 24 hours. In control explants cultured alone, BM axons, identified by SC1 immunostaining [21], followed a reasonably straight path laterally and away from the internal floor plate (Figure 3A). However, in the presence of the juxtaposed floor plate, motor axons deflected reproducibly (Figure 3B). Confocal images of immunostained explants were used to measure the angle of BM axon bundles relative to the border with the additional floor plate, and the mean angle for each explant category was determined. The mean deflection angle was 7° for control explants and 26° in the presence of the additional floor plate.

We then applied to these co-cultures antibodies to the Unc5a, Robo1/2 and Neuropilin-1 receptors, which mediate the repellent effects of Netrin-1, Slits and Sema3A, respectively (reviewed in [1]). We found that application of anti-Unc5a antibody at 10 μg/ml reduced floor plate-dependent repulsion to 12° (Figure 3C, E), significantly different from controls with floor plate, but not significantly different from controls without floor plate. Therefore, this antibody completely blocked floor plate repulsion. Application of an anti-Robo1 antibody had no effect, while anti-Robo1/2 antibodies elicited a dose-dependent reduction in repulsion at 10 μg/ml and 100 μg/ml, with the latter concentration producing complete blockade (Figure 3E). Both Robo1 and Robo2 participate in floor plate repulsion of BM axons in the rat [7], and these data suggest that both receptors are also involved in the chick. By contrast, anti-Neuropilin-1 antibodies applied at 10 μg/ml or 100 μg/ml did not significantly reduce floor plate-mediated repulsion (Figure 1E), suggesting that Sema3A plays no role. Application of anti-Unc5a and anti-Robo1/2 antibodies together at 10 μg/ml and 100 μg/ml, respectively, reduced the mean deflection angle to 8° (Figure 3D, E). This is the largest reduction in repulsion we observed, and is significantly different from controls with floor plate but not significantly different from controls without floor plate (see Additional file 1 for statistics). Taken together, these data suggest that both Unc5a and Robo1/2-dependent mechanisms mediate floor plate repulsion.

To confirm that these antibodies block the repellent effects of Netrin-1 and Slits in our culture system, we co-cultured hindbrain explants for 24 hours with their rostral/caudal borders juxtaposed to HEK293T cell clusters secreting either Slit-1 or Netrin-1 or mock-transfected (control). In co-cultures with mock-transfected cells, most clusters were permissive, with motor axons growing into them (Additional file 2A). By contrast, most Slit-1 or Netrin-1-secreting cell clusters were inhibitory, with motor axons failing to grow into them (Additional file 2B). Application of anti-Unc5a antibodies to Netrin-1 co-cultures, or of anti-Robo1/2 antibodies to Slit-1 co-cultures resulted in a significant increase in the number of clusters that were invaded by axons (permissive; Additional file 2). However, anti-Unc5a antibodies did not affect Slit-mediated repulsion nor did anti-Robo1/2 antibodies block Netrin-1-mediated repulsion. This demonstrates that the anti-Unc5a and anti-Robo1/2 antibodies block BM axonal responses to Netrin-1 and Slit, respectively.

Taken together, these experiments indicate that both Netrin-1 and Slits contribute to the floor plate repulsion of cranial BM axons. The finding that either antibody alone reduces axon deflection to control levels indicates a degree of redundancy in the function of Netrin-1 and Slit-mediated repulsive systems.

### Slit and Netrin-1-mediated growth cone collapse requires ROCK, MLCK and myosin II function

**Figure 4:**
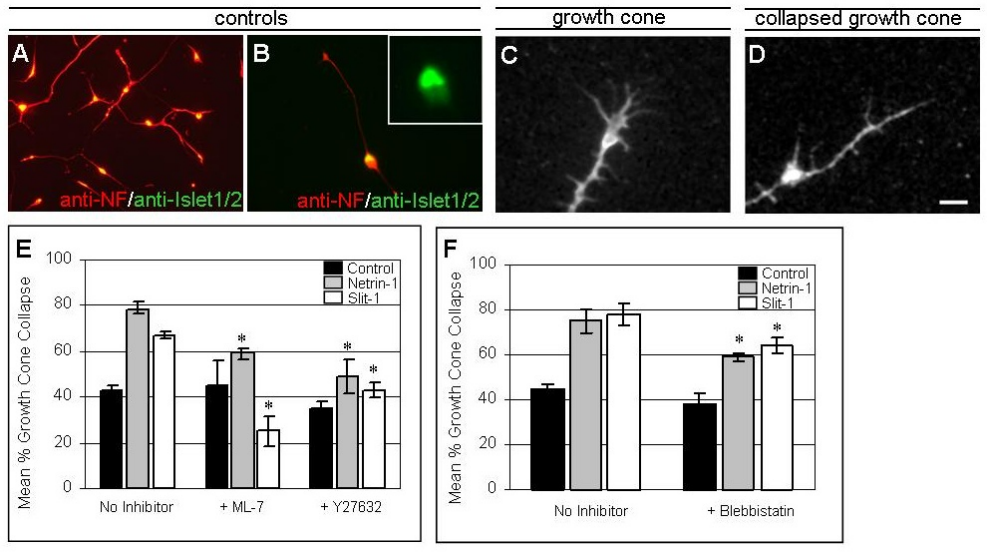
**MLCK and ROCK mediate Slit and Netrin-1-dependent growth cone collapse**. **(A-D) **Cultures of dissociated ventral hindbrain neurons. (A,B) Cultures immunostained with anti-neurofilament antibodies (red) and anti-Islet1/2 antibodies (green; single channel fluorescence image in inset). (C,D) Higher power view of growth cones immunostained using Alexafluor-568-phalloidin to show typical uncollapsed (C) and collapsed (D) morphology. In (C) the growth cone has a clear central domain, filopodia and lamellipodia, whereas in (D) the collapsed growth cone appears as a spike. **(E,F) **Histograms representing the mean percentage growth cone collapse in cultures treated with or without ligands and the inhibitors ML-7 (MLCK) or Y27632 (ROCK) or Blebbistatin (myosin II) (1 to 10 μM). Error bars indicate estimated standard mean. Scale bar = 20 μm (A), 10 μm (B) or 5 μm (C,D).

In order to investigate signalling downstream of Netrin-1 and Slits in BM neurons, we used a growth cone collapse assay [22]. The ventral third of r2 to r8 E5 chick hindbrains was dissociated into single cells, and the neurons were cultured at low density on laminin substrata for 48 hours. We found that 30 to 40% of neurons were Islet-1/2-positive motor neurons (Figure 4A, B). Although SM neurons also express Islet1/2, BM neurons are the most numerous motor neuron subtype in the hindbrain. We then quantified the responses of these motor neurons in growth cone collapse assays (Figure 4C, D). For this, medium containing purified Netrin-1 or Slit protein, or control medium, was applied alone or together with pharmacological inhibitors of ROCK, MLCK or myosin II [23,24].

The collapse rate was 43% for controls, whereas application of Netrin-1 induced 78% of growth cones to collapse. This rate was reduced to 59% by inhibition of MLCK (ML-7; Figure 4E), which was not significantly different from the collapse rate for controls, or for neurons treated with inhibitor alone (45%). This demonstrates that Netrin-dependent growth cone collapse requires MLCK. Addition of the ROCK inhibitor (Y27632) together with Netrin-1 also blocked growth cone collapse, resulting in a collapse rate of 49%, which was not significantly different from control cultures or cultures treated with inhibitor alone (35%; Figure 4E). ROCK is therefore also required for the Netrin-dependent growth cone collapse.

Slit-1-treated neurons had a collapse rate of 67%, which is significantly higher than control cultures and similar to Slit-mediated collapse rates of other neuronal types [25,26] (Figure 4E). Application of MLCK inhibitor or ROCK inhibitor both reduced Slit-1-dependent collapse to at or below control levels (rate reduced to 25% and 43%, respectively). Therefore, both the kinases MLCK and ROCK are downstream of Netrin-1 and Slit signalling in mediating cranial motor neuron growth cone collapse. It is unclear why MLCK inhibition reduces growth cone collapse to below control levels. However, we speculate that inhibiting the MLCK-dependent growth cone collapsing effects of Slit somehow unveils a second Slit activity, which promotes growth cone protrusion.

In order to further test the involvement of myosin II in cranial motor neuron growth cone collapse, we applied the myosin II inhibitor, blebbistatin. Application of blebbistatin at 1 μM did not enhance collapse beyond control levels (38% compared with 45%), but in the presence of Slit protein, blebbistatin attenuated collapse to 63% compared with 78% for Slit alone (Figure 4F). In addition, whereas Netrin-1 alone produced a collapse rate of 75%, blebbistatin reduced this rate to 58% (Figure 4F). In both cases this represented an attenuation of collapse rather than complete blockade, but nevertheless demonstrates a significant role of myosin II in Netrin-1 and Slit-mediated collapse. Higher concentrations of blebbistatin on dissociated neurons tended to increase growth cone collapse *per se*, and therefore make it problematic to test whether a higher dose might completely block collapse (data not shown).

### MLCK, ROCK and myosin II are required for cranial motor axon projections in explant cultures

**Figure 5:**
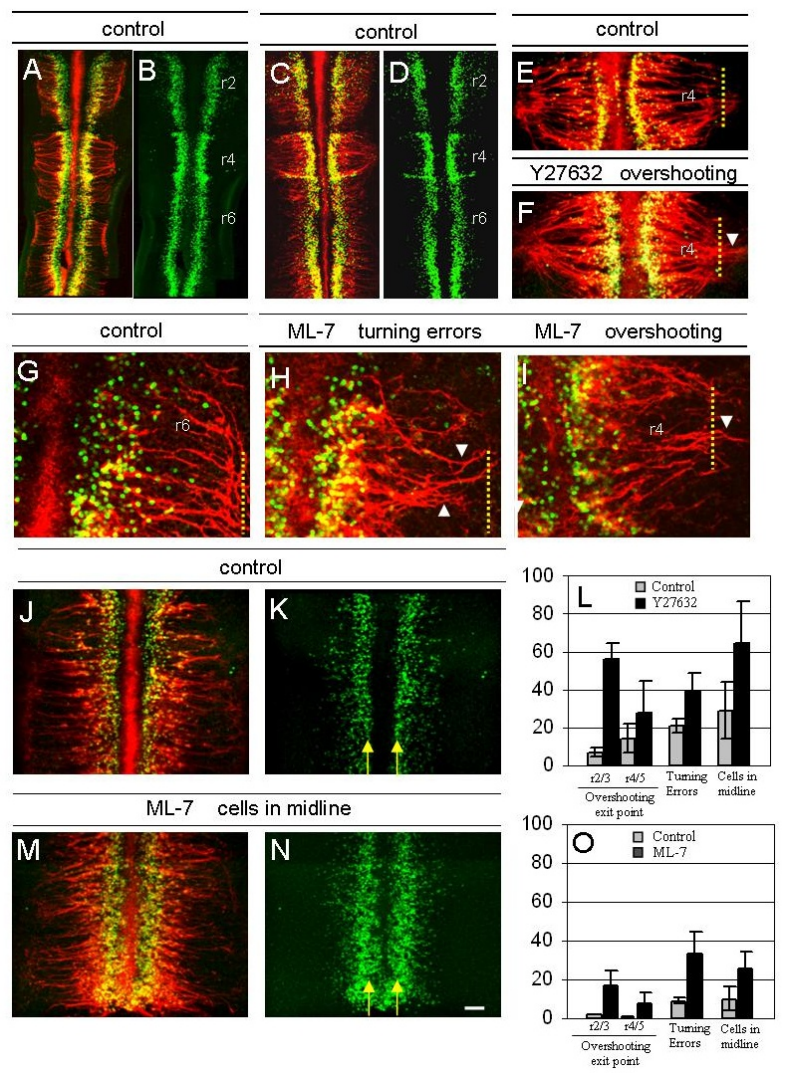
**Inhibition of ROCK and MLCK signalling causes cranial motor axon guidance defects in chick hindbrain explants**. **(A-K,M,N) **Stage 17 to 18 chick hindbrain explants cultured in collagen gels as controls or with the inhibitors ML-7 (MLCK) or Y27632 (ROCK) (10 to 20 μM), as labelled, and fixed at time 0 (A) or after 24 hours (all other panels), immunostained with anti-SC1 (red) and anti-Islet1/2 antibodies (green). In some cases (B,D,K,N) anti-Islet-1/2 immunostaining is shown as a single image of adjacent double-stained explant. (E,F,I) Rhombomere 4/5 level; (G,H) r6 level; (J,K,M,N) r6 to r8 level. Yellow arrows indicate boundaries of floor plate and arrowheads indicate axon pathfinding errors. Scale bar: (A-D) 100 μm; (E,F,J,K,M,N) 50 μm; (G-I) 25 μm. **(L,O) **Histograms representing the mean percentage of explants with three types of errors when treated with Y27632 (L) or ML-7 (O) compared with controls. Error bars represent standard error of the mean.

We next used an explant culture system to explore whether MLCK, ROCK and myosin II are involved in the elaboration of cranial motor axon pathways in the intact tissue. Stage 17 to 18 chick hindbrains (r2 to r8) were cultured as flattened preparations in collagen gels. After 24 hours *in vitro*, these explants were immunostained using anti-SC1 and anti-Islet1/2 antibodies to visualise motor axons and motor neuron cell bodies, respectively [21,27]. Immunostaining of control explants fixed at 0 and 24 hours showed that key features of BM axon pathways were present and were maintained (Figure 5A-D). BM neuron cell bodies were segregated on either side of the floor plate (Figure 5B, D) and axons in even-numbered rhombomeres projected directly to exit points, while those in odd-numbered rhombomeres made a rostral turn (Figure 5A, C).

We tested whether MLCK and ROCK are required for axon navigation by treating explants with inhibitors of these kinases and scoring for pathfinding errors. These errors were: 1, aberrant entry of cranial motor neuron cell bodies and axons into the floor plate; 2, incorrect axon projection away from the midline, including failure in the rostral turning of axons in odd-numbered rhombomeres; and 3, incorrect channelling of axons into the exit point, leading to dorsal overshooting. Errors were scored if at least one example was seen per explant; the range was usually one to five errors per explant.

The inhibitors caused no apparent changes in either the number of Islet1/2-positive cell bodies or the number of BM axons, demonstrating a lack of toxicity. However, explants treated with the ROCK inhibitor Y27632 contained motor axons that frequently overshot the exit point, and instead extended to the dorsal limit of the roof plate (Figure 5E). In control explants, BM neurons within r2/3 and r4/5 projected beyond the exit point in only 7% and 14% of explants, respectively, but this figure rose to 56% and 28%, respectively, in Y27632-treated explants (Figure 5E, F, L). Y27632 treatment also resulted in many cell bodies and axons being misplaced in the floor plate (65% compared with 29% of controls; Figure 5L) and axons from odd-numbered rhombomeres also failed to turn rostrally towards their exit points (39% compared with 21% of controls; Figure 5L). In explants treated with the MLCK inhibitor ML-7, similar types of defects were observed. Axons overshot the r2/3 and r4/5 exit points in 17% and 8% of explants, respectively, compared with 2% and 1% of control explants (Figure 5G, I, O). Turning errors in r3 and r5 also occurred in which axons failed to reach their exit points (33% compared with 9% of controls; Figure 5G, H, O), and ectopic axons or cell bodies in the midline were observed (36% compared with 10% of controls; Figure 5J-N, O). Such defects were also observed *in vivo *following attenuation of Slit-mediated repulsion [7] and following attenuation of Netrin-1 signalling (Figure 1). Thus, these three types of abnormalities can be interpreted in terms of a loss of repulsive signalling, suggesting that ROCK and MLCK transduce such signals and are required for correct BM axon navigation.

**Figure 6:**
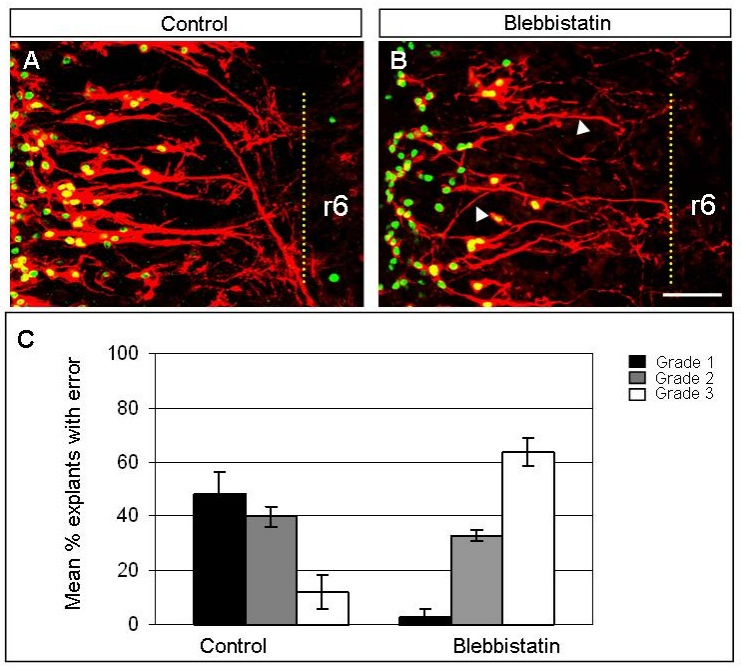
**Blebbistatin causes a loss of correct motor axon pathfinding in explants**. **(A,B) **Stage 17 to 18 chick hindbrain explants (r6/7 level) cultured in collagen gels with control medium (A) or blebbistatin (B) showing axon stalling and loss of projection from the midline. Scale bar = 100 μm. **(C) **Histogram representing mean percentage of explants showing different grades of axon pathfinding defects in controls or those treated with blebbistatin at 20 μM.

Further experiments in which the myosin II inhibitor blebbistatin was applied resulted in more profound defects in BM axon pathfinding. In contrast to the effects in explants treated with ROCK and MLCK inhibitors singly, axons in blebbistatin-treated explants stalled, wandered and failed to project away from the midline (Figure 6A, B). Due to the more severe nature of these defects, explants were scored on a scale of 1 to 3, ranging from normal patterning of axon projections (1) to a severe loss of correct projections (3). For control explants, the majority (48%) were grade 1, whereas following treatment with blebbistatin grade 3 explants comprised 64% of the population (Figure 6C). Very similar results were obtained following treatment of explants with ML-7 and Y27632 in combination (data not shown). Taken together, these data suggest that ROCK, MLCK and myosin II are required for correct BM pathfinding in the intact hindbrain, and especially for guidance away from the midline and towards exit points. As the defects observed were quite severe, whereas blebbistatin had more modest effects on Slit and Netrin-1-dependent growth cone collapse, this may imply that myosin II functions in additional signalling pathways in BM axon navigation *in vivo*.

### Deregulation of RhoA and myosin II activity *in vivo *results in cranial motor pathfinding errors

**Figure 7:**
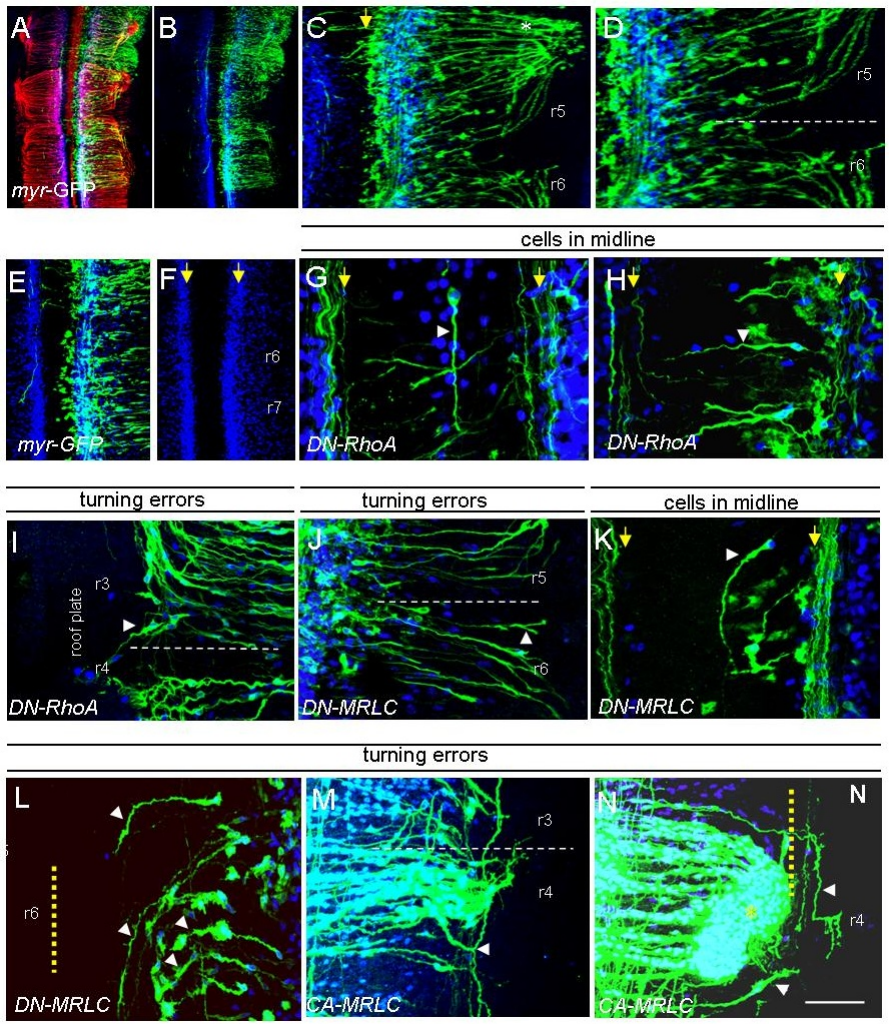
**Expression of dominant-negative RhoA, MRLC and constitutively active MRLC in chick hindbrains causes BM axon pathfinding errors**. **(A-N) **Flat-mount chick hindbrains that have been electroporated with plasmids encoding myristylated GFP (myr-GFP), dominant-negative RhoA (DN-RhoA), dominant negative MRLC (DN-MRLC) and constitutively active MRLC (CA-MRLC) as labelled (note that (A-D) are myr-GFP controls). Immunostaining was with anti-GFP (green) and anti-Islet1/2 (blue) antibodies; (A) also immunostained using anti-SC1 antibodies (red). Rhombomeres are labelled. Yellow arrows show borders of the floor plate. White dotted lines show rhombomere boundaries. Yellow dotted lines show dorsal limit of exit point. White arrowheads indicate axon pathfinding errors. Scale bar: (A,B) 200 μm; (E,F) 100 μm; (G,H,K,L) 25 μm; (H,I,K) 15 μm; (C,D,J,M,N) 50 μm.

In order to obtain *in vivo *evidence for a role of RhoA and myosin II, we electroporated dominant-negative forms of RhoA (DN-RhoA) and dominant-negative or constitutively active forms of the MRLC (DN-MRLC or CA-MRLC) into the chick embryo. These mutant forms were expressed in the chick β-actin promoter-driven construct containing an IRES-GFP, as for DN-Unc5a, with myr-GFP construct as a control. Chick embryo hindbrains were electroporated at stage 12/13 or stage 18/19, incubated for 48 or 24 hours, respectively, to stage 20/21, and immunostained for GFP, Islet-1/2, neurofilament or SC1 in various combinations. BM axon pathfinding errors were noted as for explants and DN-Unc5a electroporations. Electroporation of the control vector into ventral regions resulted in widespread expression of GFP or myr-GFP by BM neurons and no evidence of disruption to their axon pathways or cell body position (Figure 7A-F). SC1-immunostaining of electroporated hindbrains showed that BM neurons formed parallel fascicles *en route *to their exit points and that axons in odd-numbered rhombomeres - for example, r5 - turned rostrally towards their exit points (Figure 7A-D).

Cranial motor neurons that expressed DN-RhoA did not show gross perturbations in their numbers or their location; however, axons made pathfinding errors. The most obvious defect observed was a loss of repulsion from the midline, seen in 18 out of 18 electroporated embryos. Motor neurons were found in the floor plate with their axons extending longitudinally (Figure 7G), while some neurons with somata correctly located in the motor column extended axons into the floor plate (Figure 7H). This observation strongly supports the idea that RhoA mediates floor plate repulsion of cranial motor neurons and complements explant culture results, demonstrating that loss of ROCK activity results in reduced floor plate repulsion. In 6 out of 18 and 3 out of 18 embryos, respectively, axons turned aberrantly or overshot their exit points (Figure 7I; data not shown).

Electroporation of DN-MRLC produced no gross perturbations of cranial motor neuron organisation. As for DN-RhoA, loss of midline repulsion, exit point overshooting and turning errors were consistently observed. In 9 out of 16 embryos, a mean of 4 GFP+/Islet-1/2+ neurons and their axons were ectopically located within the floor plate (Figure 7K). Turning errors were observed in 8 out of 16 embryos, with axons in odd-numbered rhombomeres continuing to grow dorsally, rather than turning towards exit points (Figure 7J) or turning prematurely rather than projecting dorsally as far as the exit point (Figure 7L). In 6 out of 16 embryos, axons also overshot the exit points (data not shown). These results complement those obtained with MLCK inhibition in explants.

CA-MRLC-expressing axons showed a different repertoire of pathfinding errors. The defect in common with DN-MRLC expression was that CA-MRLC-expressing BM axons turned aberrantly towards the incorrect exit point (11 out of 18 embryos), and in 2 out of 18 cases, axons overshot the exit points (Figure 7N). BM axons were found to branch aberrantly, and in many cases projected longitudinally up and down at the level of the exit point, crossing inappropriate rhombomere boundaries (15 out of 18 embryos; Figure 7M).

Taken together, these data demonstrate that inhibition of RhoA/MRLC causes cranial motor axon pathfinding errors, consistent with a loss of midline repulsion and exit point targeting. Constitutive activation of MRLC led to exit point overshooting and overbranching in dorsal hindbrain. These results suggest that fine control of RhoA/ROCK and MRLC are required for cranial motor axon pathfinding decisions in the hindbrain.

## Discussion

**Figure 8:**
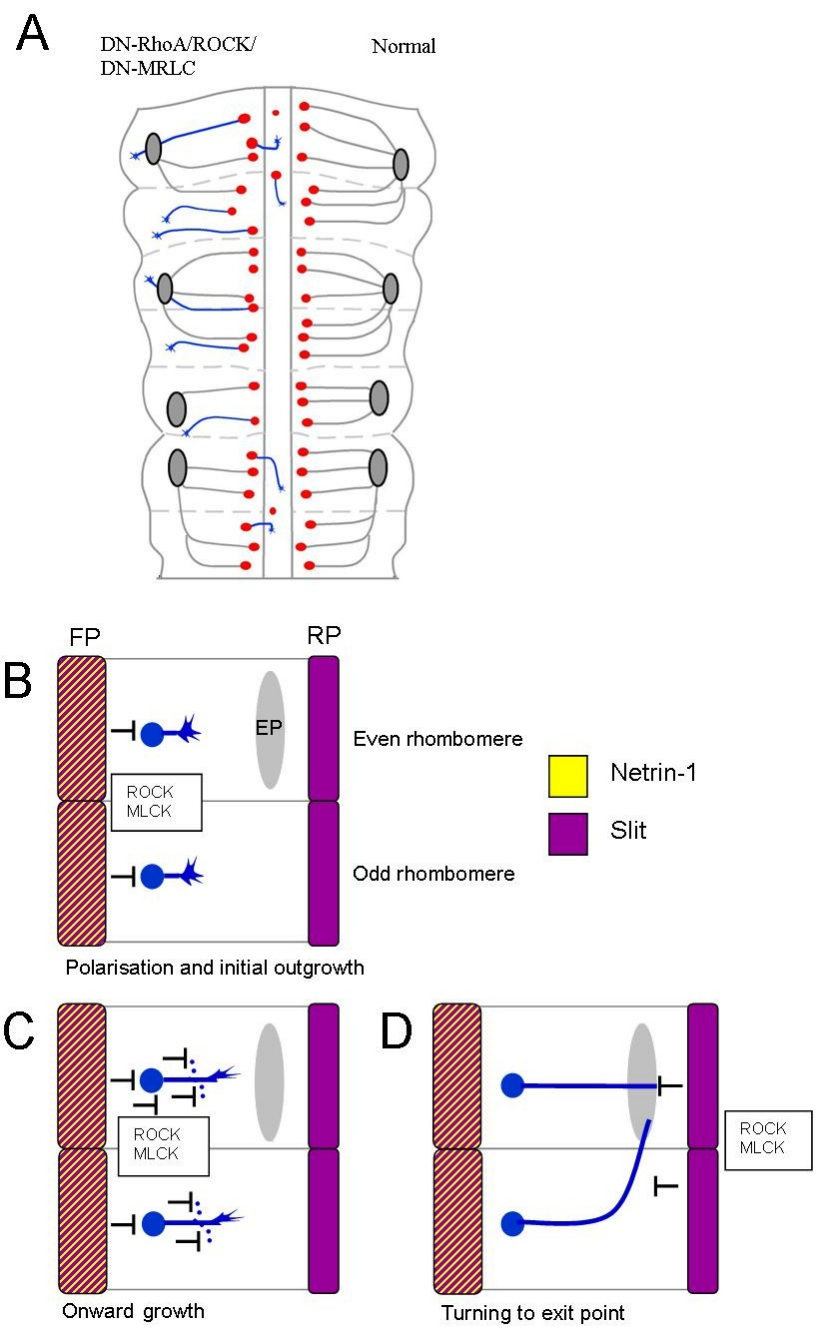
**Schematic summaries of axon guidance errors and role of repulsion in cranial motor axon pathfinding**. **(A) **Schematic diagram of chick hindbrain with grey axons on control (right) representing correct trajectories, away from the floor plate towards the dorsal exit points (grey ellipses). On the left side (pathfinding errors) blue axons represent axon guidance errors induced by loss of ROCK or MLCK function *in vitro *and loss of RhoA or MRLC function *in vivo*. **(B-D) **Schematic diagrams showing operation of repulsive mechanisms during BM cranial motor axon pathfinding in even and odd-numbered rhombomeres during (B) initial polarisation and outgrowth, (C) onward growth and suppression of ectopic branching/turning, and (D) roof plate repulsion (especially via MLCK and myosin II), which hems in axon trajectories dorsally and ensures correct axon turning towards exit points. EP, exit point; FP, floor plate; RP, roof plate.

In this paper we have demonstrated that both Slits and Netrin-1 contribute to floor plate chemorepulsion of cranial motor axons, and that Netrin-1 plays a role in this process *in vivo*. Repulsive responses of cranial motor axons to Slit and Netrin-1 require the activity of ROCK, MLCK and myosin II, pinpointing actin retrograde flow as a key executor of cranial motor neuron repulsion. Within the intact hindbrain, *in vitro *or *in vivo*, inhibition of RhoA/ROCK and inhibition or over-activation of MLCK/myosin II pathways led to reproducible defects in axon pathfinding (Figure 8). Inhibition of these candidate molecules led to the ectopic positioning of cell bodies and axons in the floor plate, and the failure of axons to project or to target exit points correctly. All of these defects are consistent with a loss of repulsion by the floor plate and/or the dorsal neuroepithelium, and closely resemble defects resulting from attenuation of Slit or Netrin-1 signalling. Therefore, Slits and Netrin-1 play key roles in BM axon repulsion, acting via ROCK, MLCK and myosin II to regulate the growth cone cytoskeleton.

### Slits and Netrin-1 collaborate in floor plate repulsion

The results of BM axon deflection assays suggest that both Slits and Netrin-1 but not Sema3A play a role in floor plate repulsion. Abrogation of either Slit or Netrin-1-dependent mechanisms was sufficient to block repulsion. Supportive evidence that both mechanisms participate comes from attenuating repulsive function in *Slit1/2 *double mutants, *Robo1 *or *2 *single mutants or *Netrin-1 *mutants ([7] and this study). In all cases, phenotypes appear to reflect a partial elimination of floor plate repulsion. There is a discrepancy, therefore, between the presence of defects in single mutants, apparently reflecting an additive effect of Slit and Netrin-1 repulsion, and the redundancy of both mechanisms in our floor plate deflection assay. We think that this might reflect technical limitations of the deflection assay, and indeed in this system blocking both Netrin-1 and Slit signalling produces the strongest effect.

It would be challenging to abrogate both Slit and Netrin repulsive mechanisms genetically. But in view of the fact that we previously found that Slit-3 was not repulsive [7], analysis of *Slit-1*/*Slit-2*/*Netrin-1 *triple mutants would therefore be very interesting. Although we found that Sema3A did not play a role in floor plate repulsion, it remains possible that Sema3A derived from the notochord might contribute to cranial motor axon repulsion from outside the central nervous system [10]. Floor plate-derived SM axon repellents remain to be identified, as neither Slits nor Netrin-1 repel these axons [6,7].

Our finding that antibodies to Unc5a and Robo1/2 block BM axon repulsion, and that *Unc5a *loss-of-function produces pathfinding defects, implicates these receptors in responses to Netrin-1 and Slits, respectively. While Robo1 and 2 have previously been established as responding to Slits in BM neurons, Netrin-1 signalling via Unc5a is less well understood. Whereas DCC (Deleted in colorectal cancer) alone can mediate Netrin-dependent attraction, Unc5a can function alone or with DCC to mediate repulsion [28-30]. We have previously shown that in the rat, *Unc5a *is expressed early during cranial motor axon projection away from the midline, while *DCC *is expressed later [13]. This suggests that Unc5a alone mediates the initial phase of cranial motor axon outgrowth; however, we have not formally tested the role of DCC.

### Slit and Netrin-1-mediated growth cone collapse requires MLCK, ROCK and myosin II

Our experiments show that ROCK (and by implication RhoA) is required for both Netrin-1 and Slit-dependent motor neuron growth cone collapse. RhoA has been proposed to be involved in repulsive signalling in several systems (reviewed in [17,18]) - for example, in Robo-dependent repulsive signalling at the midline in *Drosophila *[31] and in growth cone collapse [32]. Sema3A-dependent growth cone collapse in dorsal root ganglion neurons was found to be partially blocked by inhibition of ROCK [24]. However, a role for ROCK downstream of Netrin-1 and Slit in growth cone collapse has not previously been demonstrated. ROCK phosphorylates and activates MRLC [33,34], and our results implicating both ROCK and myosin II in growth cone collapse suggest that MRLC is an important target of ROCK phosphorylation in our system. However, ROCK can also phosphorylate LIM kinase and thence affect the activity of cofilin, which is involved in actin severing/depolymerisation (reviewed in [18]). Indeed, cofilin has been implicated as a target of Slit signalling in *Xenopus *retinal axons [26], and it remains an interesting question as to whether cofilin is involved in the repulsive guidance events downstream of Slit in BM neurons. Our evidence for the roles of MLCK and myosin II in Slit and Netrin-1 signalling is consistent with studies in *Drosophila *suggesting that MLCK acts downstream of both Netrin and Slit-dependent axon pathfinding decisions at the midline [35].

### RhoA, ROCK, MLCK and myosin II regulate cranial motor neuron pathfinding *in vivo*

Taken together, data from explant cultures and *in vivo *experiments suggest a crucial role of RhoA acting via ROCK and MLCK/myosin II in BM pathfinding decisions. In explants or *in vivo*, attenuation of either ROCK or MLCK function led to reproducible pathfinding errors that bore a close resemblance to axon pathfinding defects produced by attenuation of Slit-Robo signalling [7] (Figure 8A) and in *Netrin-1 *mutant/*DN-Unc5a*-expressing embryos. Inhibition of myosin II led to a loss of projections away from the midline, suggesting that myosin II is a crucial target of ROCK and MLCK to regulate cranial motor axon outgrowth and guidance (Figure 8).

RhoA/ROCK and MLCK/myosin II pathways thus ensure segregation of cranial motor neurons and their axon projections ipsilateral to the floor plate during normal development. Turning errors of r3/r5 axons were observed following attenuation of MLCK or MRLC function *in vitro *or *in vivo*, and axons overshot exit points. We speculate, therefore, that ROCK and MLCK act via myosin II to ensure axon deflection at the dorsal side of the neuroepithelium. Indeed, it has previously been shown that actin retrograde flow mediated by myosin II is required to suppress inappropriate protrusions on the side facing away from an attractant cue during growth cone turning [36]. Experiments demonstrating a role for MLCK and myosin II in dorsal root ganglion axons turning at borders between a laminin substratum and a non-permissive substratum support this idea [23]. Our interpretation is that BM axon turning towards exit points depends on localised repulsion at the dorsal edge of the neuroepithelium, possibly dependent on a narrow domain of Slit expression, which hems axons in to their exit points [7,12,34]. Indeed, when isolated from its adjacent mesenchyme, the dorsal tissue of the hindbrain acts as a source of chemorepulsion [37]. We cannot formally exclude, however, that attenuation of ROCK/MLCK/myosin II function blocks responses to exit point-derived chemoattraction [21]. However, the nature of such putative chemoattraction is unknown, and the most likely source, the boundary cap cells [38], are absent from our explant cultures. We also found that expression of CA-MRLC produced defects in BM axon guidance, including axons that overshot their exit points and branched ectopically. Fine regulation of MRLC activity therefore appears to be required for accurate BM axon guidance.

## Conclusions

### Model of repellent signalling pathways in cranial motor axon guidance

Our model, based on the current and previously published data, can be summarised as three phases of axon pathfinding during the dorsal projection of BM hindbrain motor neurons (Figure 8B-D). In the initial phase, Slit and Netrin-1 signalling from the floor plate, acting via RhoA/ROCK and MLCK/myosin II, would act to polarise the initial projection of motor axons away from the midline, and to exclude their axons and cell bodies from this region (Figure 8B). Secondly, the gradient of repulsion would steer axons away from the midline and suppress inappropriate branches (Figure 8C). Thirdly, a repellent border of Slit dorsally, acting via ROCK and MLCK, would mediate turning of axons in odd-numbered rhombomeres towards their exit point, and ensure the correct termination of both odd- and even-numbered axons at the correct dorsoventral level of the exit point (Figure 8D). An exit point-derived chemoattractant and/or anteroposterior polarisation of the neuroepithelium is required to explain the rostral (rather than caudal) projection. Nevertheless, key features of cranial motor axon navigation appear to depend on repulsive signalling mechanisms.

## Materials and methods

### Embryos

Fertilised hens' eggs (Winter farm or Henry Stewart farm, UK) were incubated to relevant stages [39] at 37°C.

### *Netrin-1 *mutant mice

*Netrin-1 *mutant mice were a kind gift of Dr Marc Tessier-Lavigne and were genotyped as previously described [19]. Embryos were obtained at E11.5; DiI retrograde labelling and whole-mount immunohistochemistry were performed using anti-neurofilament antibody 2H3 (Developmental Studies Hybridoma Bank, Iowa City, Iowa, USA; 1:100) as previously described [40,41]. Hindbrains were dissected out, flat-mounted and axon pathways were imaged using a laser-scanning confocal microscope.

### BM axon deflection assays

For BM axon deflection assays, ventral explants were dissected from stage 18 to 21 embryo chick hindbrains as previously described [7,42]. Floor plate explants were dissected separately and juxtaposed to the rostral or caudal hindbrain explant borders in collagen gels, in medium based on OptiMEM (Invitrogen, Paisley, UK) [7]. The antibodies used were anti-Unc5a, anti-Robo1 and anti-Neuropilin-1 (R & D Systems, Abingdon, Oxon., UK) and an anti-peptide antibody recognising Robo1 and Robo2 ('S3'; kind gift of Dr V Sundaresan) [40]. Binding to chick proteins was confirmed using western blots on hindbrain protein extracts (data not shown). Antibodies were used at concentrations of 10 to 100 μg/ml.

After 24 hours *in vitro*, gels were immunostained using antibodies to the SC1/BEN glycoprotein present on motor neurons/axons (anti-SC1 at 1:10 or anti-BEN at 1:70; Developmental Studies Hybridoma Bank) [21,43]. Secondary antibodies were Alexa Fluor-conjugated 488, 568 or 633 at 1:800 (Invitrogen, Paisley, UK) or Cy3-conjugated antibody (Stratech Scientific, Suffolk, UK). The angle of deflection of axon bundles was measured on confocal images (Scion Image programme, NIH; Figure 2B) to derive a mean axon deflection. Two angles were quantified per explant and a mean was derived for the whole population of explants in a particular condition, as it would not have been valid to choose one angle per explant.

To verify that antibodies blocked BM axon responses to Slit and Netrin, HEK293T cells were mock-transfected (control) or transfected with full-length human myc-tagged Slit expression constructs (hSlit-1; kind gift of Dr S Sakano, Asahi Kasei Corporation, Japan) in pcDNA3.1 (Invitrogen) [44] or a Netrin-1-secreting cell line was used (kind gift of Dr C Holt). Cell clusters were made in hanging drops and were co-cultured adjacent to the rostral/caudal explant borders in collagen gels for 48 hours as previously described [6,37]. Immunohistochemistry was as above and cultures were scored as to whether SC-1-positive BM axons entered the cell cluster (permissive), or avoided it (inhibitory) (Additional file 2).

### Dissociated motor neuron cultures for growth cone collapse

Glass coverslips were coated with poly-D-ornithine and laminin (15 μg/ml and 1 mg/ml, respectively; Sigma, Dorset, UK). E5 (stage 25 to 26) hindbrains were isolated as described above and the ventral third of the neuroepithelium was dissected out, removing the floor plate, in L15 medium (Gibco). Calcium and magnesium-free Hank's Balanced Salt Solution (HBSS; Invitrogen, Paisley, UK) was added for 1 to 2 minutes, replaced with 1 ml trypsin (Gibco) and incubated for 15 minutes at 37°C. Trypsin was then replaced with trypsin inhibitor solution and the tissue dissociated by triturating 15 times. The supernatant was added to 1 ml of medium containing Neurobasal medium with 2% B27 supplement, 2% horse serum, 0.1% β-2-mercaptoethanol, 0.35% Glutamax, 1% chick embryo extract, 1% penicillin/streptomycin and 50 ng/ml ciliary neurotrophic factor (CNTF) (all reagents from Sigma or Gibco). Cells were counted and resuspended for plating at 75 × 10^3 ^per coverslip in 200 μl of medium.

Axon guidance molecules and/or inhibitors were diluted in pre-warmed media and applied for 30 minutes before fixation. Both Slit and Netrin proteins (R & D Systems) were applied to neuronal cultures at concentrations of 750 ng/ml to 2 μg/ml, while inhibitors were used at 1 to 10 μM. In some experiments *Drosophila *Slit (dSlit) was transfected into HEK293 cells and the Slit protein was purified from the conditioned medium [45]. Controls were treated with normal medium, or controls for dSlit experiments were treated with conditioned medium from mock-transfected HEK293T cells. Coverslips were then fixed for 10 minutes in warmed 4% paraformaldehyde, rinsed in phosphate-buffered saline and blocked using 1% bovine serum albumin/0.5% TritonX100 in phosphate-buffered saline for 1 hour. Mouse anti-Islet1/2 (4D5; Developmental Studies Hybridoma Bank) and rabbit anti-neurofilament antibodies (AB1991; Chemicon, Millipore, Watford, UK) were then added in blocking solution for 24 to 48 hours at 4°C. Cultures were washed for 1 hour three times in blocking solution, before Cy5-conjugated goat anti-rabbit, AlexaFluor-488 anti-mouse and Alexa-Fluor-568-phalloidin in blocking solution were added overnight at 4°C. After further washes, coverslips were mounted in Fluorsave (Chemicon). At least 30 growth cones per condition were scored in three separate experiments for collapsed or uncollapsed morphology. Growth cones were only scored if they belonged to neurons containing an Islet-1/2-positive cell body, and the neuron was not in contact with any other neuron. The growth cones on axons that were at least three times the length of the cell body were scored, and a growth cone was scored as collapsed if there were fewer than four to five filopodia or if it had no clearly defined central domain. The Chi-squared test was used to compare data statistically.

### Whole hindbrain cultures

Stage 17 to 18 hindbrains (r2 to r8) were isolated, flattened and cultured in collagen gels in OptiMEM-based medium (Gibco) as previously described [37] for 24 hours. Immunohistochemistry was as above using anti-SC1/BEN antibodies in combination with the 321 polyclonal antibody to Islet-1/2 or A8 polyclonal antibody to Islet-1 (1:2,000 and 1:1,000, respectively; kind gifts of Dr T Jessell). Inhibitors used were Y27632 (ROCK), ML-7 (MLCK) and blebbistatin (myosin II) (Calbiochem, Nottingham, UK; 10 to 20 μM) [23,24]. Controls were untreated, except those for blebbistatin, which were treated with vehicle (1 μl/ml dimethyl sulfoxide; DMSO).

Explant cultures were scored for BM neuron/axon guidance defects, namely: 1, ectopic BM neurons/axons in the floor plate; 2, turning errors of r3/r5 axons *en route *to their exit points; and 3, overshooting of exit points by BM axons. For assessment of cell bodies in the floor plate, rhombomere levels 4/5 were excluded due to the contralateral vestibuloacoustic neurons, a subset of which cross the midline [46]. After blebbistatin treatment (20 μM), explants were scored on a scale of 1 to 3: 1 = normal development; 2 = intermediate development; and 3 = disrupted development, with few axons projecting away from the floor plate.

### Electroporation of chick embryos *in ovo*

Hens' eggs were incubated to stage 10 to 11 and processed after [47]. The fourth ventricle was microinjected with the appropriate DNA construct. Control constructs were *GFP *or myristylated *GFP *(*myr-GFP*), each regulated by a ß-actin promoter with a cytomegalovirus enhancer, and incorporating an IRES. Experimental constructs employed the same vector containing GFP, and included DN-Unc5a (dominant negative form lacking the cytoplasmic domain; kind gift of Dr M Tessier-Lavigne), and DN-RhoA (dominant negative form that competes with the endogenous molecule for binding to cellular guanine nucleotide exchange factors (GEFs) and cannot activate a downstream response; kind gift of Dr C Nobes) [48,49]. DN-MRLC and CA-MRLC (dominant-negative and constitutively active forms of MRLC) were also used, in which an alanine substitution for Ser19 and Thr18 results in an unphosphorylatable form, while an asparagine substitution at the same position produces a pseudophosphorylated, consitutively active form (kind gift of Dr Y Rao) [50].

Embryos were incubated for 24 or 48 hours to stage 20/21 (depending on the age at electroporation) and immunohistochemistry was performed as described previously [7], using anti-GFP (Molecular Probes, Invitrogen; rabbit or chick; 1:800), anti-SC1 mouse monoclonal (Developmental Studies Hybridoma Bank; 1:10), anti-Islet1/2 mouse monoclonal (4D5; 1:100), and anti-neurofilament rabbit polyclonal Ab1991 (1:1,000; Chemicon). Secondary antibodies and microscopy were as above.

## Abbreviations

BM: branchiomotor; CA: constitutively active; DCC: DCC, Deleted in colorectal cancer; DN: dominant-negative; E: embryonic day; GFP: green fluorescent protein; MLCK: myosin light chain kinase; MRLC: myosin regulatory light chain; myr-GFP: myristylated GFP; r: rhombomere; ROCK: RhoA kinase; Sema3A: Semaphorin3A; SM: somatic motor; VM: visceral motor.

## Competing interests

The authors declare that they have no competing interests.

## Authors' contributions

AM performed the majority of experiments in this study; other experiments were performed by AN and SB. AM had substantial input into experimental design and performed all statistical analysis, as well as contributed to writing of the paper. SG conceived the study and experimental design, took a major role in writing the paper, with collaborative input and extensive discussions with UD, who is joint grant holder.

## Supplementary Material

Additional file 1 **Statistical comparisons of effects of antibodies in floor plate deflection assay**. Click here for file

Additional file 2 **Confirmation that antibodies to Unc5a and Robo1/Robo2 block the effects of Netrin-1 and Slit, respectively**. **(A,B) **Examples of chick hindbrain explants in collagen gels (immunostained with anti-SC1 antibodies after 24 hours *in vitro*) with their rostral/caudal borders facing clusters of HEK293 cells that were either mock-transfected (A) or transfected with Slit-1 (B). Axons enter the cluster (permissive) (A) or avoid the cluster (inhibitory) (B). **(C) **Table showing effects of control and Netrin-1 or Slit-1-secreting HEK293T cell clusters in permitting or inhibiting cranial motor axon outgrowth and effects of anti-Unc5a or anti-Robo1/2 antibodies. Scale bar = 100 μm. Click here for file
