# Supplementary material for: Slit and Netrin-1 guide cranial motor axon pathfinding via Rho-kinase, myosin light chain kinase and myosin II
Source: Neural Dev. 2010 Jun 22;5:16. doi: 10.1186/1749-8104-5-16 (PMC2907369; doi:10.1186/1749-8104-5-16)
Supplement: Additional file 1 — Statistical comparisons of effects of antibodies in floor plate deflection assay. [file 1749-8104-5-16-S1.DOC]

**Supplemental Table 1: Statistical comparisons of effects of antibodies in floor plate deflection assay.**

|  | Anti-Unc5a 10 μg/ml | Anti-Robo1 10 μg/ml | Anti-Npn1  10 μg/ml | Anti-Npn1 100 μg/ml | Anti-Robo1/2  10 μ g/ml | Anti-Robo1/2  100 μg/ml | Anti-Robo1/2 10ug/ml + anti-Unc5a 10 μg/ml | Anti-Robo1/2  10 μg/ml + anti-Unc5a 100 μg/ml |
| --- | --- | --- | --- | --- | --- | --- | --- | --- |
| Control without FP | P>0.05 | P<0.00001 | P<0.00001 | P0.000001 | P<0.00001 | P<0.05 | P<0.00001 | P>0.05 |
| Control with FP | P<0.05 | P>0.05 | P>0.05 | P>0.05 | P<0.05 | P<0.00001 | P<0.05 | P<0.00001 |
