# Supplementary figures and images for: Slit and Netrin-1 guide cranial motor axon pathfinding via Rho-kinase, myosin light chain kinase and myosin II
Source: Neural Dev. 2010 Jun 22;5:16. doi: 10.1186/1749-8104-5-16 (PMC2907369; doi:10.1186/1749-8104-5-16)

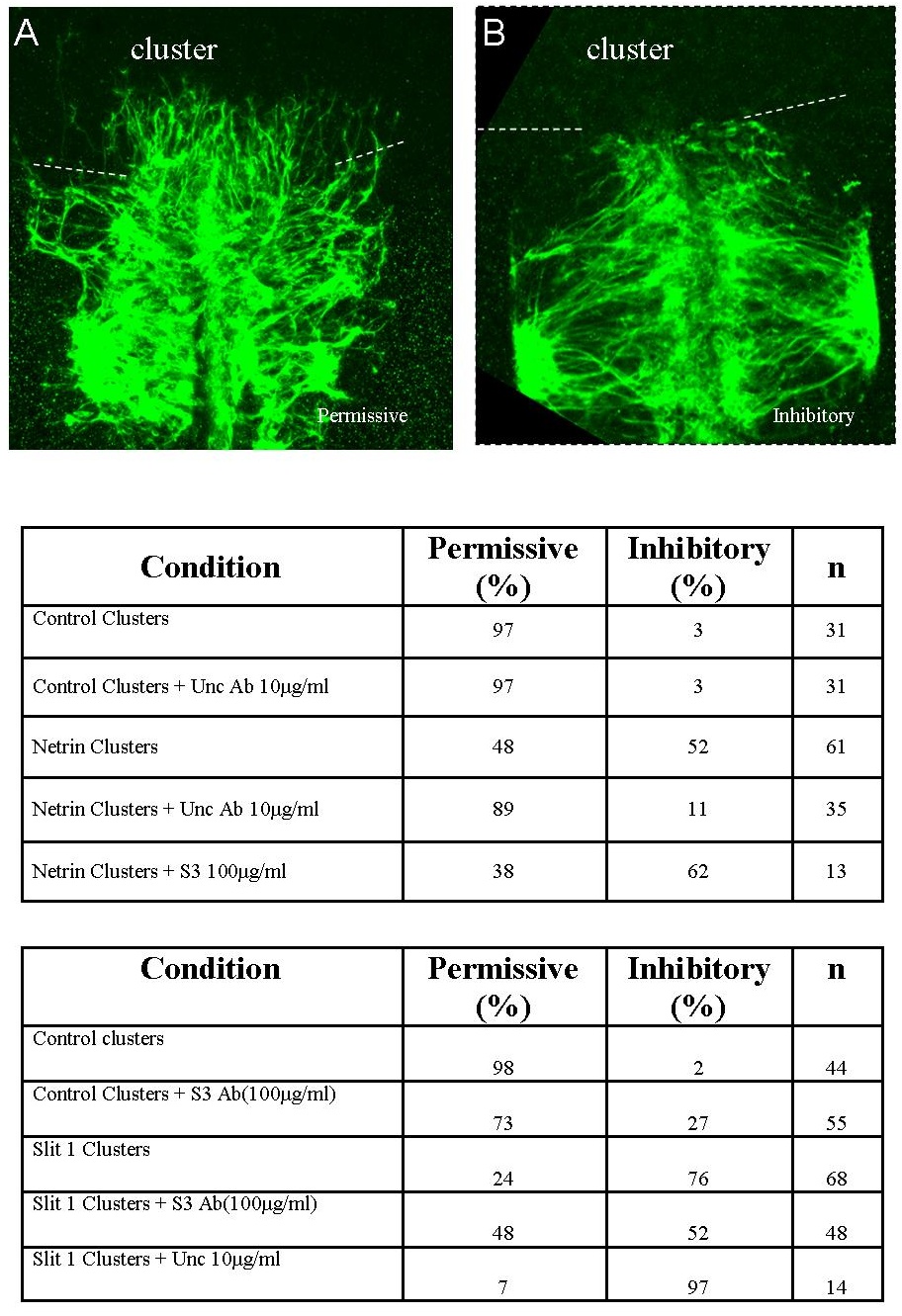

Supplement: Additional file 2 — Confirmation that antibodies to Unc5a and Robo1/Robo2 block the effects of Netrin-1 and Slit, respectively. (A,B) Examples of chick hindbrain explants in collagen gels (immunostained with anti-SC1 antibodies after 24 hours in vitro) with their rostral/caudal borders facing clusters of HEK293 cells that were either mock-transfected (A) or transfected with Slit-1 (B). Axons enter the cluster (permissive) (A) or avoid the cluster (inhibitory) (B). (C) Table showing effects of control and Netrin-1 or Slit-1-secreting HEK293T cell clusters in permitting or inhibiting cranial motor axon outgrowth and effects of anti-Unc5a or anti-Robo1/2 antibodies. Scale bar = 100 μm. [file 1749-8104-5-16-S2.JPEG]
